# The Urinary Polyomavirus-Haufen Test: A Highly Predictive Non-Invasive Biomarker to Distinguish “Presumptive” from “Definitive” Polyomavirus Nephropathy: How to Use It—When to Use It—How Does It Compare to PCR Based Assays?

**DOI:** 10.3390/v13010135

**Published:** 2021-01-19

**Authors:** Volker Nickeleit, Vicki G. Davis, Bawana Thompson, Harsharan K. Singh

**Affiliations:** Division of Nephropathology, UNC-School of Medicine, Brinkhous-Bullitt Bldg., Room 409, Campus Box 7525, 160 Medical Drive, Chapel Hill, NC 27599-7525, USA; volker_nickeleit@med.unc.edu (V.N.); vicki_davis@med.unc.edu (V.G.D.); bawana_thompson@med.unc.edu (B.T.)

**Keywords:** biomarker, biopsy, BK-virus, diagnosis, electron microscopy, histology, kidney transplantation, disease classes, urine, viral load

## Abstract

“Definitive” biopsy proven polyomavirus nephropathy (PyVN), usually caused by BK polyomavirus (BKPyV), remains a significant infection of kidney transplants. Diagnosis depends upon an allograft biopsy and outcome depends upon early intervention. Here, we report data on a non-invasive biomarker for PyVN, the urinary PyV-Haufen test. Test results were compared to those of conventional laboratory assays targeting PyV replication, i.e., BKPy-viremia, -viruria and urinary decoy cell shedding. Of 809 kidney transplant recipients, 228 (28%) showed PyV replication with decoy cell shedding and/or BKPy-viremia by quantitative PCR; only a subset of 81/228 (36%) showed “definitive” PyVN. Sensitivity and specificity for identifying patients with PyVN was: 100% and 98%, respectively, urinary PyV-Haufen test; 50% and 54%, respectively, urinary decoy cell shedding; 97% and 32%, respectively, BKPy-viremia with cut-off of ≥250 viral copies/mL; 66% and 80%, respectively, for BKPy-viremia ≥10^4^ viral copies/mL. The PyV-Haufen test showed a very strong correlation with the severity of PyVN (Spearman’s ρ = 0.84) and the Banff PyVN disease classes (*p* < 0.001). In comparison, BKPy-viremia and -viruria levels by PCR displayed modest correlations with PyVN severity (Spearman’s ρ = 0.35 and 0.36, respectively) and were not significantly associated with disease classes. No association was found between decoy cell shedding and PyVN severity or disease classes. Pilot data demonstrated that PyVN resolution with decreasing Banff pvl-scores was reflected by a gradual decrease in PyV-Haufen shedding; such a tight association was not noted for BKPy-viremia. In conclusion, urinary PyV-Haufen testing is a highly specific, non-invasive method to accurately diagnose patients with “definitive” PyVN and to optimize patient management. Assay specifics are discussed.

## 1. Introduction

Polyomaviruses (PyV) are ubiquitous, small, double-stranded DNA viruses that exist in symbiosis with man and animals. After a primary infection at a young age, PyV generally resides in a latent stage in the uro-renal tract as well as other anatomic sites of healthy asymptomatic individuals. Several human polyomavirus strains can coexist in the same host organism, and even in the same organ, such as BKPyV and JCPyV in the kidney or bladder [1]. Sero-prevalence depends on the viral strain and the patient age; it ranges from 20% to greater than 90% [2]. Latent PyV strains, alone or in combination, can undergo episodes of self-limiting asymptomatic reactivation with viruria and, on occasion, also viremia. However, despite a high prevalence of latent PyV infections and despite transient asymptomatic activation of latent PyV, manifestation of viral disease is rare. It typically develops in patients with a compromised immune status, such as renal disease/polyomavirus nephropathy (PyVN) caused by BKPyV replication in immunosuppressed kidney transplant recipients [3,4,5]. Increasing evidence also links BKPyV to carcinogenesis in the uro-renal tract post renal transplantation [2,6,7,8,9], and to hemorrhagic cystitis after bone marrow transplantation [10].

Here, we will focus on PyVN and diagnostic biomarkers with special emphasis on the urinary PyV-Haufen test.

**Figure 1:**
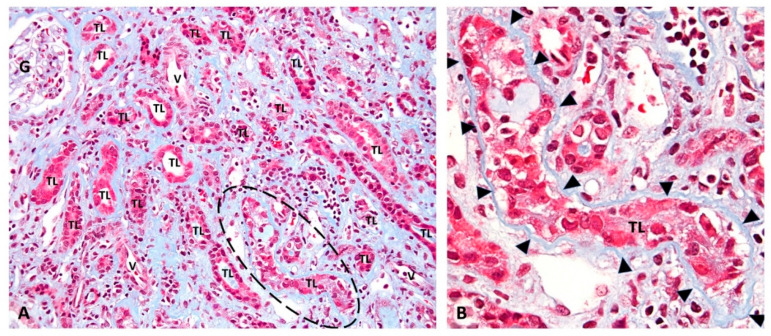
Renal injury in “definitive” PyVN. Renal biopsy: (**A**) low-power view of a representative case of PyVN demonstrating many tubular cross sections with virally induced injury. (**B**) The circled tubular cross section in “A” is shown at higher magnification. PyV replication causes marked injury to tubular epithelial cells with cell sloughing into tubular lumens (TL) and denudation of basement membranes. (**A**) Trichrome stain, 200× magnification. (G, glomerulus; TL, tubular lumen; V, blood vessel; dashed circle outlines a virally injured tubule). (**B**) Trichrome stain, 400× magnification (arrowheads, tubular basement membrane; TL, tubular lumen).

**Figure 2:**
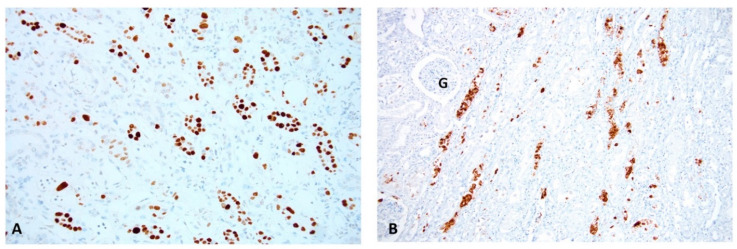
PyV replication in “definitive” PyVN. Renal Biopsy: (**A**) An immunohistochemical stain for the SV40-T antigen shows PyV replication with many positive (brown) staining signals in tubular epithelial cell nuclei. This stain targets an early PyV gene product/an intra-nuclear protein associated with PyV replication. (**B**) An immunohistochemical stain for PyV capsid protein (VP1) demonstrates strong staining (brown) for late PyV gene products. Abundant staining is found not only in nuclei, but also in tubular lumens post release of daughter virions from lysed tubular cells, (compare to Figure 2A and Figure 3). Immunohistochemistry on formalin fixed and paraffin embedded tissue sections, (**A**) antibody directed against the SV40 T antigen, 200× magnification. (**B**) antibody directed against the polyomavirus VP1 capsid protein, 100× magnification; (G, glomerulus).

Renal disease with “definitive” biopsy-proven PyVN: PyVN is characterized by morphologically apparent virally induced tubulo-interstitial injury [5,11,12]. It was first described in a kidney transplant by Mackenzie in 1978 [13]. Frank renal disease, also referred to as “definitive PyVN” [14], is defined by intra-renal PyV replication, mainly in tubular epithelial cells that typically causes host cell lysis with the release of mature daughter virions into tubular lumens (Figure 1 and Figure 2).

Thus, PyVN is a form of virally induced tubular injury that can range from minimal, i.e., PyV replication in rare tubules only, to marked, i.e., PyV replication in many tubules with marked tubular cell lysis and even interstitial fibrosis [15]. The different phenotypes of PyVN are subtyped into three disease classes reflecting the degree of viral replication and the degree of chronic injury [16,17,18]. PyVN, most commonly found in renal allografts and only seldom found in native kidneys [19], is mainly caused by the replication of BKPyV (in >98% of cases), rarely by JCPyV and hardly ever by SV40PyV [20,21,22,23]. Diseased renal allograft recipients are usually immunosuppressed with a tacrolimus-based drug regimen that seems to provide a window of opportunity for PyV replication [12,24,25,26,27]. The incidence of PyVN varies among transplant centers; it usually ranges between 4% and 6% in western countries. Viral nephropathy can lead to allograft dysfunction and graft loss, the latter seen in 8% to 30% of PyVN patients within 24 months (dependent upon the era of transplantation) [16,17]. Targeted and effective anti-PyV/PyVN therapy is currently not available. Outcome depends on an early diagnosis when virally induced renal injury is limited, and intervention with a reduction in the immunosuppressive therapy is most effective. The overall prognosis is best in early stages of disease and worst in late stages with chronic kidney injury [16,17].

Patients with PyVN do not show symptoms of a generalized infection or an active urine sediment, and allograft dysfunction may be absent. More than 50% of transplant recipients in disease class 1 and over 25% in disease class 2 present with stable serum creatinine levels at time of diagnosis [16], Thus, the clinical timing and indication of a diagnostic renal biopsy can be challenging.

Clinical patient management: Over the last two decades, adjunct urine and plasma-based laboratory assays have been developed to identify patients at increased risk for PyVN and to guide patient management post kidney transplantation [14,28]. Testing is based on the paradigm that “practically” all patients with PyVN present with signs of PyV replication/activation, including viremia and viruria. Conversely, only a subgroup of patients with viral replication are, indeed, diseased, or will develop disease [29]. Thus, while the negative predictive value of “PyV-replication-based laboratory tests” to exclude PyVN from the list of differential diagnoses is generally high, the overall positive predictive values are relatively low, and the diagnosis of “definitive” PyVN with kidney injury requires an invasive renal biopsy. In the context of patient screening, quantitative plasma PCR tests to evaluate BKPy-viremia/BKPyV-DNAemia levels are routinely utilized and widely accepted; urine cytology for the detection of PyV inclusion bearing decoy cells or urine-based PCR testing for BKPy-viruria is less commonly used [11,20,28,30,31,32,33].

Viremia levels with a cut-off of 10^4^ BKPyV copies (DNA gene equivalents)/mL plasma or greater have a higher positive predictive value for underlying “definitive” PyVN [20]. Conventionally, such viremia levels are considered “high risk” and classified as “presumptive” PyVN if additional confirmatory renal biopsy findings are lacking [14,28,34]. Often without further confirmation by biopsy, immunosuppression in viremic patients is preemptively lowered in order to prevent disease progression and the (potential) development of “definitive” PyVN: one laboratory parameter, i.e., plasma PCR data, guides therapeutic intervention, i.e., the lowering of immunosuppression.

This clinical practice is intriguing since it aims to prevent PyVN/disease and to avoid costly and invasive renal biopsies, [35,36], yet the caveat is that preemptive lowering of immunosuppression is not standardized and does not reliably prevent the subsequent development of “definitive” PyVN [37,38,39]. Furthermore, potential suboptimal baseline immunosuppression in kidney transplant recipients might increase the risk of rejection, including the development of de-novo donor-specific antibodies [39,40,41]. BKPy-viremia testing by quantitative PCR, the mainstay laboratory assay to guide preemptive clinical intervention, has limitations: (1) PCR assays and units are not standardized and inter-laboratory quantitative test results can vary significantly; (2) the PCR assays target BKPyV, whereas PyVN due to JCPyV or other PyV strains typically remains undetected; (3) BKPy-viremia can originate from extra renal tissue sites, e.g., the urinary bladder or salivary glands, and, therefore, it may not be an accurate marker of intra-renal viral disease; (4) in pediatric patients, BKPy-viremia may reflect a primary infection not associated with kidney injury or disease; (5) less than 50% of patients with viremia develop “definitive” PyVN; (6) “definitive” PyVN can be seen with low viremia of <500 BKPyV copies/mL, and conversely no PyVN may be encountered with high titers >10^4^ copies/mL.

Thus, conventional clinical decision making and preemptive therapeutic strategies with lowering baseline immunosuppression in patients with signs of PyV replication/activation are based on variables and assumptions that may or may not be best practice on an individual case basis. Is there an alternative to this “one-size-fits-all-approach”, a personalized strategy for patient management? The specific identification of diseased patients in PyVN classes 1 or 2, that typically have a favorable long-term prognosis, would be optimal [16,17]. However, such approach is hampered by the need for invasive renal biopsies.

**Figure 3:**
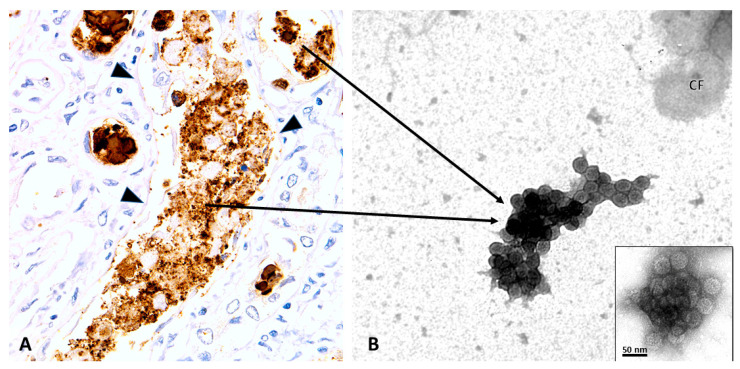
PyVN, intra tubular viral aggregation and urinary PyV-Haufen. Renal Biopsy (**A**) and Voided Urine Specimen (**B**). (**A**) Immunohistochemistry with an antibody directed against the PyV-VP1 capsid protein showing intra tubular viruses (brown) released into an injured tubule post host cell lysis. Note: denudation of tubular basement membranes. It is here that viruses form dense three-dimensional aggregates, seen as granules of varying sizes by light microscopy (LM). PyV aggregates are subsequently flushed out of the kidney and can be found in the urine as PyV-Haufen by negative staining EM. (**B**) EM showing characteristic PyV Haufen in a voided urine sample. These PyV Haufen can be easily identified based on the uniform size of the virions and the capsid surface structure (inset). Note the three-dimensional cast-like shape of the PyV Haufen (compare to Figure 4). (**A**) Immunohistochemistry on formalin fixed and paraffin embedded tissue sections with an antibody directed against PyV-VP1 capsid protein, 400× magnification; (arrowheads, tubular basement membrane; arrows, pointing from the “birthplace” of PyV aggregates to a typical PyV Haufen seen by EM in a voided urine sample in (**B**). (**B**) EM with negative staining/uranyl acetate stain, 80,000× magnification, transmission electron microscopy; inset with 120,000× magnification (CF, cell fragment).

The urinary PyV-Haufen test: The PyV-Haufen test differs fundamentally from PCR-based assays and the detection of decoy cells, in that this test is renal-disease-specific. Haufen (Haufen is a German term for heap or pile) are three-dimensional, cast-like PyV aggregates that form within the kidney, that is, within tubules. Subsequent to PyV replication and assembly in tubular cell nuclei, virions are released from lysed host cells into injured tubules with low flow of primary urine and high concentrations of uromodulin/Tamm–Horsfall protein (Figure 2B). The aggregation of PyV depends upon high concentrations of uromodulin that serve as an “adhesive glue-like substance”, and it is very similar to the formation of other intra-tubular casts, such as red blood cell casts. Once the PyV aggregates are flushed out of the kidney, they can be identified as so-called PyV-Haufen in voided urine samples by electron microscopy (EM) and serve as specific biomarkers for “definitive” PyVN (Figure 3).

This article provides details on how to use the urinary PyV-Haufen test, when to use it, and how it compares to common PCR-based assays. In what way can patient management, including costly and invasive renal biopsies, be optimized and paradigms of PyVN be adjusted?

## 2. Main Body

### 2.1. PyV-Haufen—Definition and Characteristics

PyV-Haufen are detected in voided urine samples by negative staining EM; they are too small in size to be detectable by LM. PyV-Haufen are defined as discrete, tightly clustered, three-dimensional, cast-like aggregates of at least six distinct virions (Figure 3B) [42,43,44].

**Figure 4:**
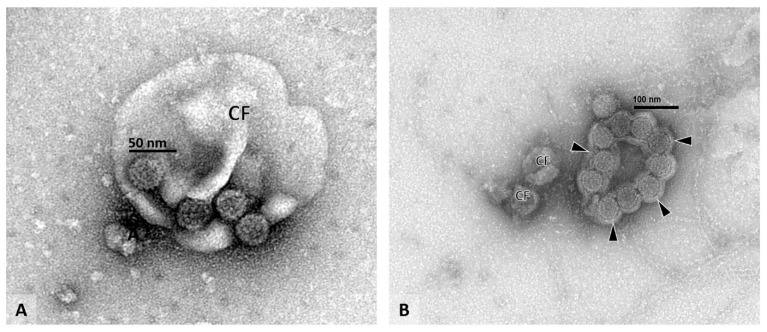
Voided urine sample with individual virions. Voided urine specimen (**A**,**B**), EM: (**A**) Four individual polyomaviruses can be seen clinging to the surface of a cell fragment (CF). Note the typical viral capsid structure and the uniform size of the viral particles measuring approximately 40 nanometers in diameter. (**B**) A flat sheet of polyomaviruses can be seen covered by a thin layer of cell membrane material (arrowheads). The characteristic viral capsid structure is visible on each virion. Since tight three-dimensional PyV aggregates are not present, the illustrated findings in (**A**,**B**) do not represent PyV-Haufen (compare to Figure 3B). (**A**) EM with negative staining/uranyl acetate stain, 100,000× magnification, (CF, cell fragment). (**B**) EM with negative staining/uranyl acetate stain, 80,000× magnification, (CF, cell fragment).

The ultrastructural morphology of PyV seen by negative staining EM is illustrated in Figure 3B and Figure 4. Individual virions are highly uniform, with a diameter of approximately 40–50 nanometers (in fixed urine samples) and a typical surface structure composed of 72 VP1 pentons. Based on morphology and ultrastructural appearance, different PyV strains cannot be distinguished.

On average, PyV-Haufen contain between 10–20 individual polyomaviruses but can occasionally be composed of more than 100 virions. Large PyV-Haufen resemble casts (Figure 3B). By definition Haufen must not contain other core components such as debris, cell membrane fragments or vesicles. However, debris including remnants of cell membranes can occasionally adhere to the outer edges of Haufen. Single virions and two-dimensional flat sheets of PyV are not classified as Haufen (Figure 4). Note: the individual size of a PyV-Haufen does not carry any diagnostic significance; in contrast, the number of PyV-Haufen per ml urine tightly correlates with the degree of virally induced intra-renal injury (see below in Section 2.2.2).

Detailed technical guidelines on PyV-Haufen testing are provided in Section 2.4 and in the Supplementary Material.

### 2.2. Urinary PyV-Haufen Assays: A Qualitative, Quantitative and Comparative Test Analysis

All patient-based data collection and analyses were approved by the UNC institutional review board (IRB).

#### 2.2.1. PyV-Haufen Testing: Qualitative Assay

We analyzed the predictive value of PyV-Haufen testing for the diagnosis of “definitive” PyVN in 809 adult renal allograft recipients transplanted at UNC between 2009 and 2019. Dependent upon the date of transplantation, patients were followed according to local standard of care guidelines for various timespans ranging from 6 months to 10 years. During the post-transplantation course, the activation/replication of PyV was assessed using urine cytology/decoy cell counts, PCR-based assays, to quantitatively screen for BKPy-viremia or -viruria and PyV-Haufen tests following local clinical assessment and published guidelines [14,45]. Allograft biopsies were collected in cases of graft dysfunction or BKPy-viremia of ≥250 viral copies (gene eqivalents)/mL plasma. “Definitive” PyVN was diagnosed in biopsies according to published criteria [15,16,17,18]. For the current study purposes, patients were sub-grouped based on their PyV activation/replication status, as assessed for each patient over the entire available individual post transplantation follow-up period:

1. Group 1, no PyV activation: 581/809 patients (72%):
  - Decoy cell analysis negative or with no more than one positive decoy-test
    (cut-off for positive test ≥10 decoy cells per ThinPrep cytology preparation);
  - BKPy-viremia undetectable or always less than 250 viral copies/mL plasma;
  - No “definitive” PyVN in all available biopsies.
2. Group 2, PyV activation with positive decoy-cell tests: 37/809 patients (5%):
  - Decoy cell analysis positive with two or more positive decoy-tests
    (cut-off for positive test ≥10 decoy cells per ThinPrep cytology preparation);
  - BKPy-viremia undetectable or always less than 250 viral copies/mL plasma;
  - No “definitive” PyVN in all available biopsies.
3. Group 3, PyV activation with low level BKPy-viremia: 78/809 patients (10%):
  - Decoy cell test positive or negative;
  - BKPy-viremia between 250 and 9999 viral copies/mL plasma
    in one or more tests, and no test ≥10^4^ viral copies/mL plasma;
  - No “definitive” PyVN in all available biopsies.
4. Group 4, PyV activation with high level BKPy-viremia: 32/809 patients (4%):
  - Decoy cell tests positive or negative;
  - BKPy-viremia variable with at least one test ≥10^4^ viral copies/mL plasma;
  - No “definitive” PyVN in all available biopsies.
5. Group 5, biopsy proven “definitive” PyVN: 81/809 patients (10%):
  - Decoy cell tests positive or negative;
  - BKPy-viremia variable;
  - Biopsy diagnosis of PyVN in all patients.

Post transplantation, 228/809 (28%) patients presented with some form of PyV/BKPyV activation/replication (groups 2–5) and 81/809 (10%) with “definitive” PyVN (group 5). A total of 98% of patients in group 5 had detectable BKPy-viremia ≥250 viral copies/mL plasma (in two patients, plasma PCR tests were falsely negative due to presumed mutations in the VP1 region targeted by the assay). In the five groups, 210 patients were tested for urinary PyV-Haufen shedding, with emphasis on those presenting clinically with signs of PyV/BKPyV replication (Table 1). A total of 82/210 patients tested PyV-Haufen positive, 80/82 (98%) in group 5 with biopsy-proven PyVN. In contrast, 128/210 patients tested PyV-Haufen negative, 127/128 (99%) in groups 1–4, lacking a diagnosis of “definitive” PyVN.

**Figure 5:**
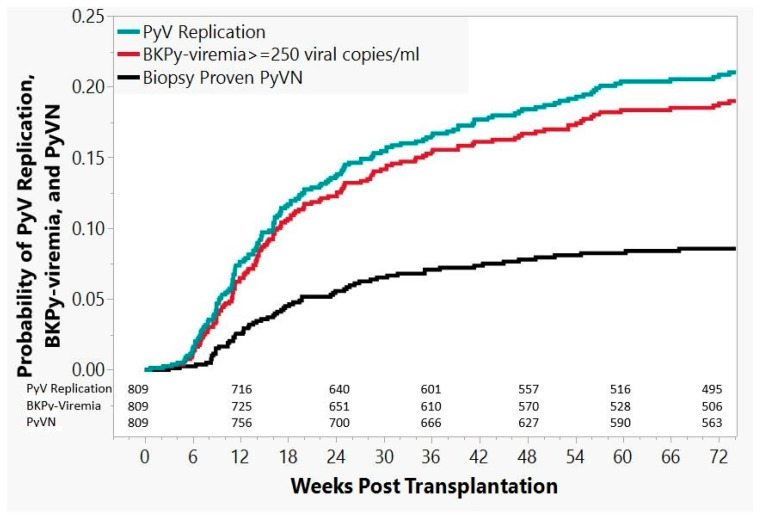
Kaplan–Meier estimates. Kaplan–Meier estimates of overall PyV replication, BKPy-viremia, and “definitive” biopsy proven PyVN in 809 kidney transplant recipients followed for 74 weeks post transplantation, when 80% of PyVN cases had developed. Numbers of patients “at risk” (i.e., neither having presented with the monitored event nor lost to follow-up) are given for each category by time point. Detailed definitions are provided above.

In the cohort of 81 patients with “definitive” PyVN, the diagnosis was established by biopsy, on average, 47 weeks post grafting (range: 3–320 weeks), with 64/81 (80%) of PyVN diagnoses established within the first 74 weeks. For this time window of 74 weeks after transplantation, Kaplan–Meier estimates are presented in Figure 5, with probabilities for overall PyV activation/replication, BKPy-viremia, and “definitive” biopsy-proven PyVN: probability of PyVN 9% (95% CI: 6.8–10.8%), probability of BKPy-viremia 19% (95% CI: 16.3–22.0%), and overall probability of any form of PyV activation/replication (decoy cell shedding and/or BKPy-viremia and/or “definitive” PyVN) 21% (95% CI: 18.2–24.1%).

A comparative analysis of binary classification tests was conducted in a sub-cohort of 182 patients, with available test results for PyV-Haufen and decoy cell shedding and/or BKPy-viremia collected in a time window of 74 weeks post transplantation (Table 2). For patients with or without “definitive” PyVN, the PyV-Haufen test had a sensitivity of 100% and a specificity of 98%. In comparison BKPy-viremia with a cut-off of ≥250 viral copies/mL by PCR had a high sensitivity of 97%, but a low specificity of 32%. Raising the cut-off in the viremia test to ≥10^4^ viral copies/mL increased the specificity to 80% but decreased the sensitivity to 66%.

#### 2.2.2. PyV-Haufen Testing: Quantitative Assay

Quantitative urinary PyV-Haufen test results were analyzed in a group of 73 adult renal allograft recipients with an established biopsy-proven diagnosis of “definitive” PyVN, who were transplanted at UNC between 2001 and 2016 (these patients were also included in previous studies [16,17]). Index biopsies collected at time of initial diagnosis were considered. Histologic findings were recorded according to Banff criteria. They included scoring results of the percentage of tubules with morphologic evidence of PyV replication (the Banff pv load/pvl-score), and the PyVN-disease classes [16,17,18,46,47]. All biopsies fulfilled Banff adequacy criteria [47,48]. Data on BKPy-viremia, BKPy-viruria (both by PCR), urinary decoy-cell shedding (urine cytology) and urinary PyV-Haufen shedding were collected within a time window of +/− two weeks of index biopsy. The aim was to correlate the degree of PyV-Haufen shedding with the severity of PyVN, i.e., the Banff pv load, and the PyVN disease classes. For comparative purposes, the same correlations were made using test results of viremia, viruria and decoy cell shedding. How accurately do tests reflect the severity of “definitive” PyVN?

The overall significance testing was conducted using the Kruskal–Wallis test. Correlations were calculated using the Spearman Rank procedure. Due to the skewed nature of the PyV-Haufen and PCR data, ranks were determined for these values and used in the plots so that linear regression lines could be calculated. For the three PyVN disease classes, test marker results are shown as medians and interquartile ranges (IQRs).

**Figure 6:**
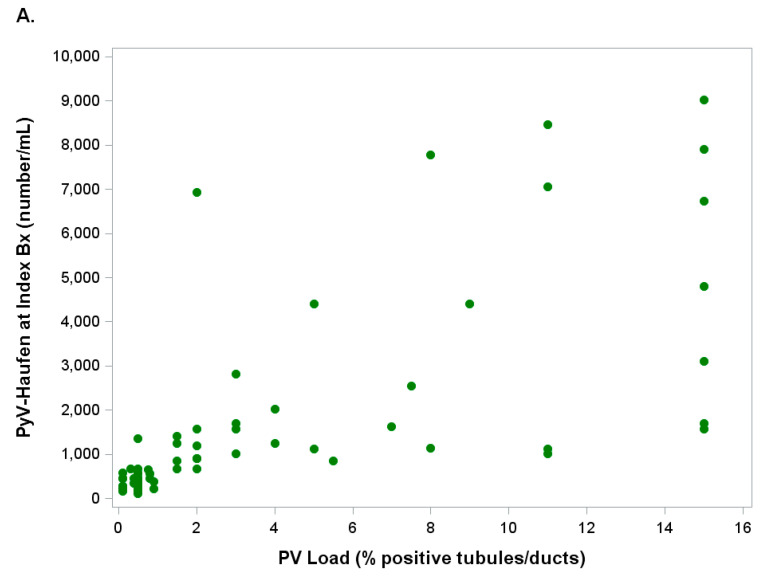

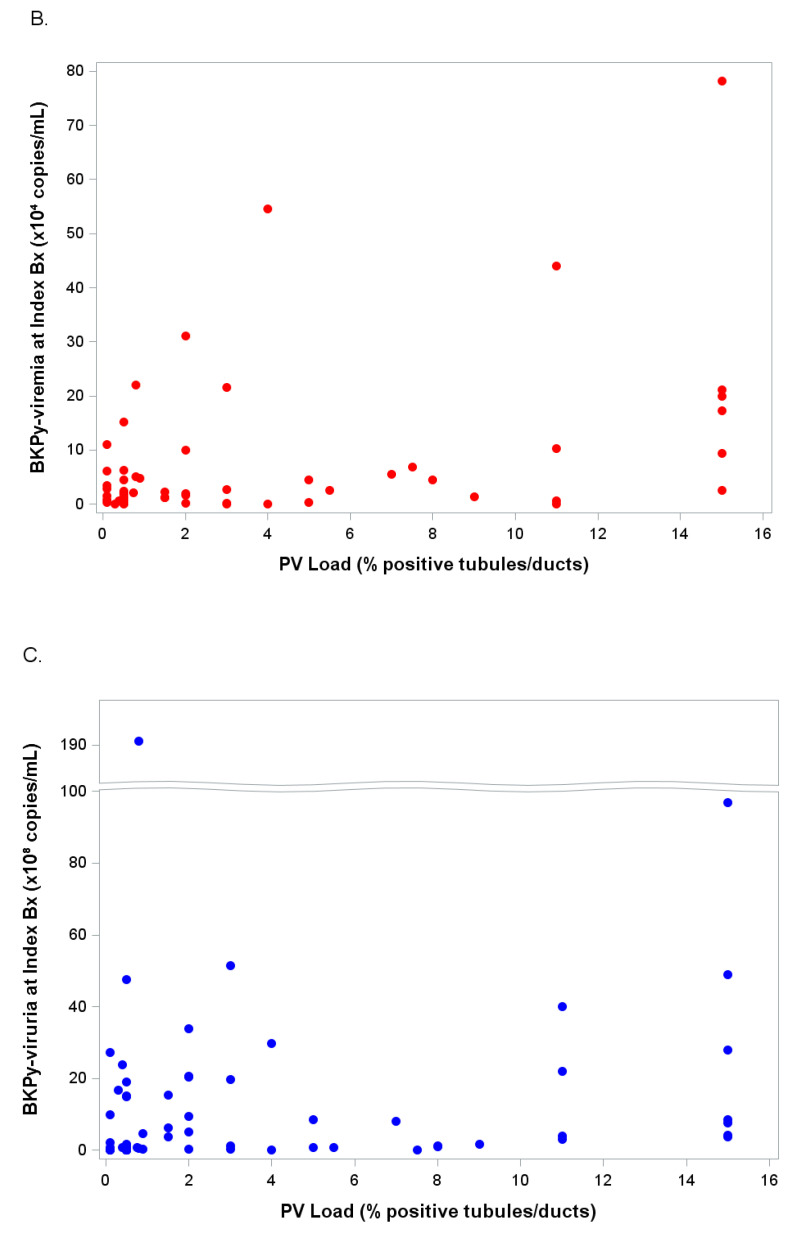
(**A**–**C**): Scatter Plots. Illustrated are quantitative marker test results (y-axis) and PyVN disease severity (percentage of virally injured tubules/Banff pv load based on SV40-T expression by immunohistochemistry) in index biopsy (x-axis). For clarity, the x-axis is truncated at the 16% mark since >98% of index biopsies showed a pv load ≤16%. (**A**) PyV-Haufen shedding (green); (**B**) BKPy-viremia (red); (**C**) BKPy-viruria (blue).

Scatter plots reflecting the relationship between quantitative test results of viremia, viruria, PyV-Haufen shedding and the Banff pv load (percentage of virally injured tubules) in corresponding biopsy tissue are illustrated in Figure 6A–C.

**Figure 7:**
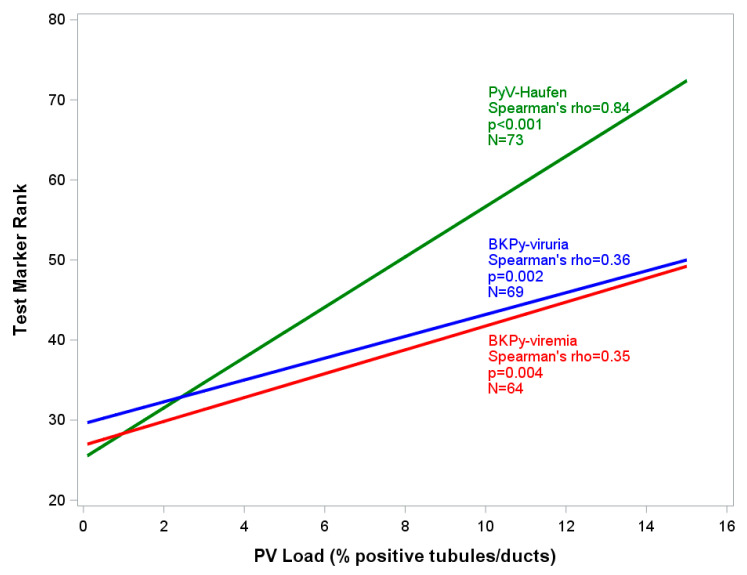
Severity of PyVN and corresponding test marker expression. Rank linear regression modelling the relationship between quantitative marker test results (y-axis) and PyVN disease severity (x-axis). Y-axis: PyV-Haufen: # per mL urine; BKPy-viremia: quantitative PCR test with viral gene copy equivalents/mL plasma; BKPy-viruria: quantitative PCR test with viral gene copy equivalents/mL urine. For individual test results, ranks were used in place of actual test values. X-axis: percentage of virally injured tubules/Banff-pv load in index biopsy based on SV40-T expression by immunohistochemistry. For clarity, the x-axis is truncated at the 16% mark, since >98% of index biopsies showed a pv load <16%. N lists the number of samples available for testing in each of the three marker categories. Green: PyV-Haufen shedding; blue: BKPy-viruria; Red: BKPy-viremia.

While PCR-based viremia and viruria test results varied broadly when plotted against the pv load, that correlation was much tighter for quantitative PyV-Haufen testing. Spearman rank correlations between test markers and percentage of virally injured tubules/pv load showed coefficients of 0.84 for PyV-Haufen testing, 0.36 for BKPy-viruria and 0.35 for BKPy-viremia (Figure 7). When quantitative PyV-Haufen test results were correlated with BKPy-viremia, the Spearman correlation coefficient was ρ = 0.36 (*n* = 64 patients, *p* = 0.004); a slightly stronger association was found for BKPy-viruria, ρ = 0.42 (*n* = 69 patients, *p* < 0.001).

A quantitative PyV-Haufen test result also proved to be highly predictive for the PyVN disease class (*p*-value < 0.001; Table 3). In contrast, the PyVN disease class could not be reliably predicted by viremia and viruria levels (*p*-values between 0.09 and 0.1, respectively, Table 3). Urine cytology and quantitation of decoy cell shedding did not correlate with the severity of PyVN or with the disease classes.

**Figure 8:**
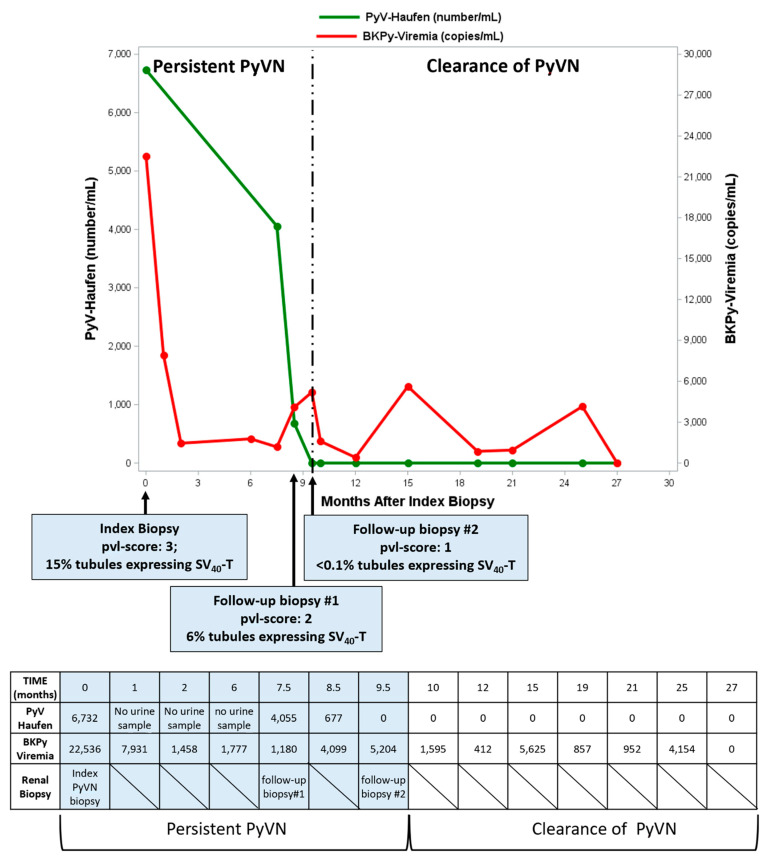
PyV-Haufen and BKPy-viremia test results during follow-up post diagnostic index biopsy. The degree of “definitive” PyVN decreased with a reduced percentage of tubules expressing SV40-T antigen (follow-up biopsy 1), and disease healed 9.5 months post diagnosis (follow-up biopsy 2). In parallel PyV-Haufen shedding decreased, ceased at time of healing and remained negative thereafter. Conversely, after an initial decrease, BKPy-viremia remained detectable at various levels over an extended period of time; BKPy-viremia levels did not mark PyVN healing.

Quantitative PyV-Haufen testing is also suited for monitoring PyVN progression or regression during follow-up. Figure 8 illustrates test data from one seminal patient post-diagnostic index biopsy. PyV-Haufen shedding gradually decreased, along with biopsy-proven viral nephropathy, and ultimately ceased 9.5 months post index biopsy when PyVN healed. All subsequent PyV-Haufen tests over months remained negative. In contrast, BKPy-viremia initially decreased but subsequently remained positive beyond PyVN healing (at levels ranging from 412 to 5625 BKPyV gene equivalents/mL plasma; manuscript in preparation).

### 2.3. Urinary PyV-Haufen Testing: Clinical Indications

Although the PyV-Haufen test was primarily validated for detecting PyVN in renal allografts, the test works equally well for viral nephropathy in native kidneys since prerequisites for Haufen formation, that is, high-intra-tubular uromodulin concentrations, are universal kidney-specific characteristics independent of transplantation, gender, ethnicity or age [49,50]. Since different PyV strains share structural characteristics, such as size and surface composition, all cases of PyVN, whether caused by BKPyV, JCPyV, or possibly SV40PyV replication [22,23], can be identified. Thus, in a case of JCPyV-induced PyVN, PCR assays targeting BKPyV can be negative while a urinary PyV-Haufen test renders positive results. Pilot studies also suggest that monitoring for disease evolution during persistent PyVN can be optimized, since longitudinal quantitative PyV-Haufen test results can provide data on changes in the severity of intra-renal viral disease. Of note: the Haufen test is not suited to diagnose BKPyV associated hemorrhagic cystitis post bone marrow transplantation (since PyV aggregates do not form in the bladder that contains low uromodulin concentrations) or to diagnose BKPyV-induced uro-renal carcinomas (due to the lack of intra-neoplastic PyV replication) [6,7,8,51]. If urinary PyV-Haufen are found in the latter patient cohorts, then an additional diagnosis of concurrent “definitive” PyVN with intra-renal lytic PyV replication has to be made [49].

Due to the nature of the PyV-Haufen test requiring an EM, it is not suited as a mass-screening assay (see below). Rather, it should be used as a targeted diagnostic tool in patients at increased risk for “definitive” PyVN, i.e., those presenting with BKPy-viremia or viruria or urinary decoy-cell shedding. The PyV-Haufen test is especially beneficial for individuals who cannot easily undergo diagnostic renal biopsy, including pediatric patients or those at risk of bleeding post hematopoietic stem cell transplantation. Furthermore, the PyV-Haufen test can be used to confirm and diagnostically validate a “PyVN negative biopsy result” (when tissue samples are small and limited) or to monitor patients longitudinally including disease progression or regression.

### 2.4. Urinary PyV-Haufen Testing: Technical Guidelines and Recommendations

Detailed protocols can be found in the Supplementary Material.

#### 2.4.1. Urine Collection and Storage

Voided urine is collected following standard protocols for cytology evaluations. The second morning, mid-stream urine is ideal. Diluted samples (post large fluid consumption) or samples collected after extended bed rest (e.g., first morning urine) should be avoided. Urine samples can be transiently kept at room temperature for approximately 30 min, but should be fixed expeditiously with freshly made 4% paraformaldehyde (ratio 1:1). In our experience, fixed urine samples are stable when stored at ambient temperature for 72 h, and for years at 4–8 °C with excellent PyV preservation. Of note, only fresh 4% paraformaldehyde should be used for preservation of PyV capsid proteins, essential for the proper ultrastructural identification of PyV-Haufen. Formalin should not be used for fixation, as the PyV capsid proteins are degraded rendering the proper recognition of virions impossible. Unfixed urine samples can be frozen at −80 °C and stored long term with good viral preservation (in our experience, from months to years).

#### 2.4.2. Grid Preparation for EM and Negative Staining Protocols

In urine samples, the number of PyV-Haufen varies from none (no PyVN) to abundant (PyVN with histologic Banff pvl-score of 3). Fixed urine samples are prepared using a 4-step method to significantly enhance EM analyses by generating cleaner and more concentrated specimens. Step 1: Sample aliquots are first cleared of large impurities such as cellular debris and membrane fragments by spinning the samples at low centrifugation to pellet out the debris. Step 2: The supernatant is subsequently filtered through a 5 µm filter. Of note, sample clarification does not lead to any appreciable loss of PyV-Haufen since their average diameter is approximately 160–700 nm (thus, much smaller than the filter-pore size of 5 µm). Furthermore, the overall low mass prevents PyV-Haufen from being pelleted/eliminated in the clarification centrifugation step 1. Step 3: Critical to the success of negative staining EM and for the detection of PyV-Haufen is sample concentration. Although PyV-Haufen can be identified in fresh untreated urine samples, the possibility of false negative readings increases if sample concentration steps are omitted. This is of particular importance in patients with limited PyVN in disease class 1/with a pvl-score of 1, in whom intra-renal disease is focal [16,17]. A number of techniques have previously been described in order to effectively concentrate viruses for EM analyses, including sucrose and cesium chloride gradient ultra-centrifugation. For routine clinical purposes, however, we concentrate the clarified urine samples by ultra-centrifugation at 20,000 *g* (12,500 rpm) for 35 min. This approach works very well since PyV-Haufen, in contrast to single virions, have a higher overall mass and are, therefore, more easily pelleted. Step 4: After ultra-centrifugation, 150 µL of the pellet (containing concentrated PyV/PyV-Haufen) is kept and used for subsequent EM grid preparation. The supernatant is discarded. Of note, in our experience, analyzing over 1000 urine samples from both “man and mice”, there is no evidence that the ultra-centrifugation step results in artificial PyV aggregation and erroneous in-vitro PyV-Haufen formation, skewing test results.

For EM analysis, formvar/silicone dioxide coated copper grids are used. To ensure a smooth, even spread of the sample and stain, grids must be rendered hydrophilic by glow discharge immediately prior to use. The grid is then incubated at ambient temperature on 30 µL (of the 150 µL) pelleted sample collected in step 4, followed by two quick dips of the grid in deionized water. Subsequently, the grid is stained with 2% uranyl acetate and allowed to air dry. Grids are ready for immediate ultrastructural evaluation. Alternatively, they can be archived for future batch evaluation in an EM grid storage box and kept in a dessicator chamber in the dark for a period ranging from days to months. Of note, this approach was developed with help from the Centers for Disease Control (CDC), using and adapting CDC technical protocols for viral identification.

#### 2.4.3. PyV-Haufen Detection by EM: Best Practice Recommendations

EM grids are examined on a standard transmission electron microscope. We recommend the systematic scanning of 25 grid squares at 50,000× magnification to screen for the possible presence of PyV-Haufen. Their definitive identification requires higher magnification (80,000–100,000×) and the unequivocal detection of three-dimensional cast-like aggregated polyomaviruses displaying the characteristic viral capsid substructure (see Section 2.1. above for PyV-Haufen definition). PyV-Haufen are typically found along with varying numbers of single virions and occasional flat, two-dimensional sheets of virions (Figure 4B). For clinical purposes, specimens are generally qualitatively categorized as either “PyV-Haufen-positive or negative” (since Haufen originate in virally injured renal tubules, even one characteristic PyV-Haufen marks renal disease). Positive urine samples usually contain several Haufen of various sizes, and these are commonly identified within the first 5–10 min of grid examination. In PyVN disease class 1/in cases with a pvl-score of 1, however, urinary PyV-Haufen are less abundant. We, therefore, recommend a total EM evaluation of 30 min per grid in order to confidently render a diagnosis of “PyV-Haufen negative”.

When examining EM grids, the detection of individual virions or flat sheets of viruses serves as a valuable indicator of viral activation. They also serve as an important internal quality control for an adequate undilute urine sample [52]. The probability of PyV-Haufen positivity rises to approximately 20% if individual free virions are noted in the background.

A *quantitative* analysis is performed by counting the total number of PyV-Haufen in 25 randomly selected grid squares, requiring, on average, approximately 30 min of scoping [43,53] The number of PyV-Haufen per ml urine is then calculated using a standard formula for the quantitation of viruses in body fluids

Number of PyV–Haufen per mL urine =

(1) $$\frac{total\;number\;PyV–Haufen}{25\;EM\;grid\;squares}\times \frac{whole\;EM\;frid\;area}{area\;of\;25\;EM\;grid\;squarea}\times \frac{\frac{150\mu l^{1}}{30\mu l^{2}}}{5^{3}}$$

Formula for quantitation of viruses from body fluids (also see Supplementary Material for details). ^1^ Volume of urine/pellet after ultracentrifugation (step 4 in Section 2.4.2). ^2^ Volume used for EM grid preparation from pellet in (1). ^3^ Volume of starting urine sample at clarification step 2 (see Supplementary Materials; note that number is calculated by the subtraction of 5mL of the added fixative from the total starting volume of 10 mL).

The total time from receipt of a voided urine specimen to a completed EM grid examination is approximately 2–3 h. Multiple samples and multiple grids (in our experience up to six) can be prepared in parallel and subsequently analyzed in batches. EM scope time per grid varies, ranging from approximately 10 min for a qualitative assessment of PyV-Haufen positivity to 30 min for a quantitative assessment or assessment of a PyV-Haufen-negative sample. Of note, degenerated urine samples with abundant debris or blood in the background (such as those seen with first morning urine collection or in patients with hematuria) or highly diluted samples (such as those seen after large fluid intake) are unsuited for proper PyV-Haufen analysis. These samples should be clearly marked as “inadequate” and a new urine sample obtained for evaluation.

## 3. Conclusions

Viral infections with latency establishing double-stranded DNA viruses are relatively common and generally remain clinically insignificant. In healthy individuals, they occasionally undergo self-limiting asymptomatic cycles of replication, while “frank disease” is rare and limited to immune compromised patients. Polyomaviruses fall into this category, with the BKPyV strain causing PyVN in immunosuppressed kidney transplant recipients. Patient management and clinical work-up for diseases caused by latency establishing viruses, such as PyVN, are challenging. Which laboratory test reflects an asymptomatic viral infection, and, on the other hand, what test result indicates disease with organ injury?

The backstay for risk assessment of PyVN has been assays targeting PyV activation/replication. This approach is governed by the paradigm that viral disease requires viral replication. Thus, negative predictive values for such tests are high: no evidence of PyV replication, very low probability of PyVN [5,14,42]. However, the specificity and positive predictive values of tests are diagnostically challenging. Generally, biopsies are required to diagnose “definitive” PyVN with renal injury, and diagnostic hurdles are high.

Here, we describe a different approach using urinary PyV-Haufen testing to diagnose “definitive” PyVN. In a 10-year observational time window, we followed 809 kidney transplant recipients, 28% of whom presented with some evidence of PyV replication/activation, i.e., urinary decoy cell shedding, and/or various levels of BKPy-viremia. Renal disease with “definitive” PyVN, however, was only diagnosed in a subset of 36% of “PyV-replicators”. The sub-cohort of patients with viral nephropathy was accurately identified by urinary PyV-Haufen testing, with a sensitivity and specificity of ≥98%. In comparison PCR assays targeting BKPy-viremia at cut-off levels of 10^4^ viral copies/mL plasma, commonly used as a laboratory test indicator for “presumptive” PyVN, showed sensitivity and specificity of 66% and 80%, respectively. Lowest sensitivity was noted for urinary decoy cell shedding. In addition to accurately identifying diseased patients with “definitive” PyVN, quantitative PyV-Haufen test results tightly reflected the severity of renal disease with a correlation coefficient of 0.84. Quantitative test results were also predictive of the recently defined three Banff PyVN disease classes. Such predictions could not be made based on BKPY-viremia levels, showing only a modest correlation with PyVN disease severity, and no correlation with the disease classes, or based on the quantification of decoy cell shedding. These results further support our previously made observations [29,42,43].

Little is known about marker expression during persistent PyVN, and how accurate test results might reflect disease progression, regression and healing. Previously, we showed that patients with biopsy-proven clearance of PyVN ceased to shed PyV-Haufen, while BKPy-viremia remained detectable in some individuals [42]. Here, we provide quantitative PyV-Haufen “pilot data” from one patient, followed longitudinally over many months. Under a reduction in immunosuppression, the degree of BKPyV induced tubular injury decreased, and, finally, PyVN healed after 9.5 months. PyV-Haufen shedding gradually decreased in parallel and turned negative at time of healing. Subsequently, PyV-Haufen test results remained negative. Conversely, BKPy-viremia remained positive after PyVN healing at intermittently high levels of >1000 BKPyV gene equivalents/mL plasma. Further detailed prospective studies are underway to better characterize test results during persistent and healing PyVN.

The accuracy of the PyV-Haufen test for identifying or excluding “definitive” PyVN from the list of differential diagnoses seems to be astonishing. The explanation for this is found in the very nature of the test, i.e., it is based on disease-specific intra-renal structural changes with uromodulin modulated intra-tubular PyV aggregation [50]. Such changes only occur in PyVN. The PyV aggregates are flushed into the urine, where they can be detected as so-called PyV-Haufen. Thus, the genesis of Haufen is very similar to that of other casts, such as red blood cells casts, carrying high predictive values for intra-renal injury and disease.

The PyV-Haufen test offers new venues for patient management. First and foremost, it allows for a non-invasive accurate distinction between patients with renal disease from those without. This includes patient cohorts presenting with high viremia, conventionally classified as “presumptive” PyVN [14]. Consequently, “presumptive” and “definitive” PyVN, that have often been lumped and treated as one disease entity, can be accurately separated, and the term “presumptive” possibly even avoided. How do patients with renal disease and “definitive” PyVN differ from those only presenting with PyV replication and high viremia? How can treatment modalities be adjusted? The PyV-Haufen test will facilitate targeted future studies. Furthermore, the test optimizes clinical management during persistent PyVN and longitudinal follow-up, since pilot data suggest that disease severity and response to therapy can be monitored accurately. In the past, such patient monitoring has been difficult using conventional PCR-based assays since viremia and viruria levels wax and wane, clouding diagnostic decision making. The PyV-Haufen test will also reduce the need for invasive renal biopsies in certain circumstances. Compared to invasive biopsies, the PyV-Haufen test is cheaper, less burdensome on patients, easily repeated on new urine samples, and it does not carry any patient risk. Since urine represents a “secretion-product” of the entire kidney, false negative test results, as encountered with small renal biopsies, are uncommon in our experience.

However, the PyV-Haufen test does have some limitations, mainly arising from the need for a transmission electron microscope, the time commitment and the level of EM expertise required for the proper test evaluation. Thus, the test is unsuited for mass screening, but rather serves as a targeted second-line assay in patients with evidence of PyV replication and increased risk for PyVN, and first and foremost, in those with high BKPy-viremia.

In summary, we describe clinical and technical specifics of the urinary PyV-Haufen test. We compare test results with those of other conventional laboratory assays and highlight its strength in diagnosing PyV induced renal disease, also referred to as “definitive” PyVN. The test will allow for an improved diagnosis and, thereby, further our understanding of biological and clinical differences between patients presenting with replicative cycles of PyV versus those with organ injury and viral nephropathy.

## Figures and Tables

**Table 1:** Urinary PyV-Haufen test results in cohorts of kidney transplant recipients with varying clinical signs of PyV replication/activation.

Groups N (Patients in Group)	PyV-Haufen Positive N (%)	PyV Haufen Negative N (%)	N Patients Tested for PyV-Haufen
**Group 1 N = 581** No PyV activation	1 (6)	16 (94)	17
**Group 2 N = 37** PyV activation with positive decoy-cell tests	0 (0)	24 (100)	24
**Group 3 N = 78** PyV activation with low level BKPy-viremia	1 (2)	56 (98)	57
**Group 4 N = 32** PyV activation with high level BKPy-viremia	0 (0)	31 (100)	31
**Group 5 N = 81** Biopsy proven “definitive” PyVN	80 (99)	1 (1)	81
N Patients tested	82	128	210

Group definitions are provided above. N refers to the number of patients.

**Table 2:** Comparative Analysis of Binary Classification Tests ^1^.

Test	Patients with “Definitive” PyVN N ^2^	Patients with “Definitive” PyVN -and- Positive Test ^3^ N	Sensitivity (%)	Patients without “Definitive” PyVN N	Patients without “Definitive” PyVN -and- Negative Test ^4^ N	Specificity (%)
**Urine cytology/Decoys**						
≥2 tests with ≥10 cells	60	30	**50**	114	62	**54**
**BKPy-viremia by PCR**						
≥10^4^ viral copies/mL	61	40	**66**	102	82	**80**
≥250 viral copies/mL	61	59	**97**	102	33	**32**
**Urinary PyV-Haufen**	64	64	**100**	118	116	**98**

^1^ Cohort is limited to 182 patients with available PyV-Haufen test results -plus- either additional test results for decoy cell shedding and/or BKPy-viremia. The overall prevalence of PyVN in this cohort is 35%. ^2^ A patient is listed as “positive” if ≥ two samples tested positive for decoy cell shedding above threshold or ≥ one sample tested positive for BKPy-viremia above threshold or ≥ one sample tested positive for PyV-Haufen shedding. All test results collected between transplantation and index biopsy were considered in the group of 64 PyVN patients. ^3^ A patient is listed as “negative” if all available test results were negative or below threshold. ^4^ All test results collected within 74 weeks post transplantation were considered in the group of 118 patients without “definitive” PyVN.

**Table 3:** Test marker expression by PyVN disease class (at time of index biopsy).

Test Marker (At Time of Index Biopsy)	Statistic	PyVN Disease Class (In Index Biopsy)			
		Class 1	Class 2	Class 3	*p*-Value *
**PyV Haufen**	**Median**	416.5	1411	7881	<0.001
**(number/mL urine)**	**IQR**	282–451	903–3104	6732–9030	
	**N**	32	39	2	
**BKPy-viremia**	**Median**	1.39	2.74	11.83	0.098
**(** **×10^4^ viral copies/mL)**	**IQR**	0.31–3.48	0.59–10.02	2.52–21.13	
	**N**	27	35	2	
**BKPy-viruria**	**Median**	0.82	5.70	3.84	0.086
**(** **×10^8^ viral copies/mL)**	**IQR**	0.28–14.90	0.80–20.70	3.60–4.08	
	**N**	29	38	2	

* Kruskal-Wallis test for Class 1 versus Class 2 (Class 3 excluded due to small case number) PyVN disease classes defined according to the Banff classification [16,17,18].

## Supplementary Materials

The following are available online at https://www.mdpi.com/1999-4915/13/1/135/s1, Supplementary Material: Detailed protocols.

## Abbreviations

BKPyV-	BK-polyomavirus strain
EM-	(transmission) electron microscopy
LM-	(standard) light microscopy
PCR-	(quantitative) polymerase chain reaction
PyV-	polyomavirus
Pvl-	histologic Banff score of intra renal polyomavirus load levels ranging from “0” (no PyV detected) to “3” (marked PyV load with >10% infected renal tubules)
PyVN-	polyomavirus nephropathy (biopsy proven definitive renal disease)
UNC	The University of North Carolina at Chapel Hill.
