# Supplementary material for: The Urinary Polyomavirus-Haufen Test: A Highly Predictive Non-Invasive Biomarker to Distinguish “Presumptive” from “Definitive” Polyomavirus Nephropathy: How to Use It—When to Use It—How Does It Compare to PCR Based Assays?
Source: Viruses. 2021 Jan 19;13(1):135. doi: 10.3390/v13010135 (PMC7833404; doi:10.3390/v13010135)
Supplement: Supplementary file 1 [file viruses-13-00135-s001.pdf]

Supplementary Material with detailed protocols:

All protocols have been developed over more than a decade based on negative staining EM analyses of over 1000 urine samples. The goal has been to establish robust reproducible procedures suited for routine use. Listed below are detailed protocols essential for successful PyV-Haufen analysis.

1) **Prepare 4% Paraformaldehyde pH 7.4** (can be stored at 4°C for 3-4 weeks)

Mix the following reagents in a 4:3:1 ratio:

- 0.2M Sorensen's phosphate buffer pH 7.2 (EMS),
- deionized water
- 32% paraformaldehyde solution (EMS)

pH to 7.4 with 10N NaOH, filter sterilize through a 0.22µm PES vacuum filtration unit

2) **Sample Preparation**

Fixation: In a 50ml conical tube fix urine samples 1:1 with 4% paraformaldehyde

Clarification step 1: Spin 12mls of fixed urine samples 1,500rpm for 5min. Decant 10mls sample supernatants into collection cups; discard pellets containing debris.

Clarification step 2: Aspirate sample supernatant with 10ml syringe, lock on a 5.0µm filtration unit and slowly push supernatant into a 9/16 x 31/2 polypropylene (polyallomer) ultracentrifuge tube

Concentration step 3: Spin samples at 12,500rpm for 35 min; gently aspirate and discard sample supernatant; leave approximately 150µl of sample pellet at bottom containing PyV-Haufen. Note: pellets may only be barely visible!

3) **EM Grid Preparation**

Glow discharge (Pelco easiGlow) EM grid support films, removable Formvar, SiO type A, 300 mesh Cu (Ted Pella), shiny side up for 2min

Dislodge sample pellet containing PyV-Haufen (from concentration step 3) by pipetting vigorously up and down many times (do not vortex!!)

Place 30µl of sample on 'staining trough' (or parafilm) – position 1 - followed by one drop of deionized H_2O each in position 2 and position 3- and 30µl of 2% uranyl acetate – position 4 (all positioned in a row)

Use Dumoxel 03 N5SP tweezers (EMS) to place EM grid shiny side down on top of sample droplet for 5min.

Use Nichrome loop (1.5mm Ted Pella) to transfer grid onto each water droplet, then to 2% uranyl acetate stain for 60 seconds.

Lift grid by its outer edge off stain with Dumoxel tweezers, and invert grid (shiny up).

Use a 1” X 0.5” inch piece of Whatman 3MM chromatography paper to wick the stain off from the surface of the grid.

Let the grid dry for 5min before analyzing by TEM or storing in an EM grid storage box, in a desiccator, in the dark.
